# Using HIV Transmission Networks to Investigate Community Effects in HIV Prevention Trials

**DOI:** 10.1371/journal.pone.0027775

**Published:** 2011-11-16

**Authors:** Joel O. Wertheim, Sergei L. Kosakovsky Pond, Susan J. Little, Victor De Gruttola

**Affiliations:** 1 Department of Pathology, University of California San Diego, San Diego, California, United States of America; 2 Department of Medicine, University of California San Diego, San Diego, California, United States of America; 3 Department of Biostatistics, Harvard University, Cambridge, Massachusetts, United States of America; Centro Nacional de Microbiología - Instituto de Salud Carlos III, Spain

## Abstract

Effective population screening of HIV and prevention of HIV transmission are only part of the global fight against AIDS. Community-level effects, for example those aimed at thwarting future transmission, are potential outcomes of treatment and may be important in stemming the epidemic. However, current clinical trial designs are incapable of detecting a reduction in future transmission due to treatment. We took advantage of the fact that HIV is an evolving pathogen whose transmission network can be reconstructed using genetic sequence information to address this shortcoming. Here, we use an HIV transmission network inferred from recently infected men who have sex with men (MSM) in San Diego, California. We developed and tested a network-based statistic for measuring treatment effects using simulated clinical trials on our inferred transmission network. We explored the statistical power of this network-based statistic against conventional efficacy measures and find that when future transmission is reduced, the potential for increased statistical power can be realized. Furthermore, our simulations demonstrate that the network statistic is able to detect community-level effects (e.g., reduction in onward transmission) of HIV treatment in a clinical trial setting. This study demonstrates the potential utility of a network-based statistical metric when investigating HIV treatment options as a method to reduce onward transmission in a clinical trial setting.

## Introduction

Randomized trials of preventive measures against HIV, such as male circumcision [1]–[3], vaginal microbicide gel [4], pre-exposure prophylaxis (PrEP) [5], [6], and vaccination [7], have demonstrated modest but potentially important benefits. In studies to date, such interventions have had estimated efficacy levels of 30–50% in preventing individual infections. What remains to be quantified, however, is the potential such interventions have for larger community-level effects (i.e. reduced infectiousness leading to lower disease burden). It is unknown whether any current intervention strategy could meet the public health goal of bringing an epidemic under control.

Prevention and treatment efforts [e.g. imperfect vaccination, PrEP followed by antiretroviral treatment (ART), or test-and-treat] could reduce onward transmission by decreasing the viral load of infected individuals [8]. Even interventions that were ineffective or not intended to prevent infections (e.g. test-and-treat [9]) may prove useful in lowering HIV's capacity for future infection by reducing viral load [10], [11]; such effects would likely be of great benefit to the susceptible population as a whole. It is noteworthy that single or two-drug ART combinations are sufficient for substantial reductions in maternal-child transmission, even though such therapies are not adequate for complete suppression of HIV [12]. Current clinical trials are not designed to detect a decrease in onward transmission as an effect of an intervention at the community level. Although group-level randomized trials have been undertaken [13], such studies are logistically complex and not always practical. This manuscript addresses the question of how to use putative transmission network information inferred from individual-level randomized studies to learn about the community-level impact of prevention strategies.

HIV prevention trials face a combination of formidable obstacles: relatively low efficacy (around 30%) and a low incidence of HIV infections (≤1% per year) in most populations. This combination often results in relatively low statistical power (<80%) to detect efficacious intervention, even for very large numbers of trial participants (∼10000 per arm). The recent CAPRISA microbicide trial in South Africa is an exception, which was able to conduct the study in a population with an unusually high incidence of HIV (around 10%) [4].

Statistical tests used to detect the efficacy of prevention and intervention treatments take into account the time of infection of study subjects but not the evolutionary transmission history of the virus. HIV is a measurably evolving pathogen, and changes in its genetic sequence over time have been successfully used to reconstruct recent individual-to-individual transmission histories [14]–[20]. These transmission histories are traditionally represented as phylogenetic trees, but they can also be depicted as (incomplete) transmission networks. These networks are comprised of nodes, representing HIV-infected individuals, which are connected by edges if the genetic similarity or phylogenetic relatedness of the viruses is sufficiently high [17], [21]. Individual nodes in a transmission network can be described using a variety of statistics, one of which is degree: the number of edges connecting to a given node.

Here we propose a statistical metric that accounts for the evolutionary relatedness of the virus and address two questions: (i) can transmission networks provide a basis for developing more powerful statistical metrics to measure prevention effeciveness, and (ii) can these transmission networks be used to detect decreases in viral transmission from study participants to others in their sexual network? Through simulations on a transmission network inferred from men who have sex with men (MSM), we demonstrate how our network metric can be more powerful than current methods in a clinical trial setting. In principle, clinical trials using this network statistic should be able to detect a decrease in secondary transmission, even if treatment fails to prevent primary infection.

## Methods

### Ethics statement

Written informed consent was obtained from all patients, and the human experimentation guidelines of the US Department of Health and Human Services were followed in conducting this research. The US Department of Health and Human Services has also issued a Confidentiality Certificate to all studies of the University of California, San Diego involving acute and early HIV infection and recruitment of sexual partners. Collection of these data was approved by the University of California, San Diego Institutional Review Board. The sequence data were anonymized.

### Construction of the transmission network

Genetic distances among HIV sequences were calculated using *pol* fragments sampled from N = 502 HIV-infected MSM from the San Diego, California area between 1996 and 2009. All samples were obtained during acute and early stages of infection. To construct the transmission network, a connection (i.e. edge) was placed between any two individuals (i.e. nodes) whose viral sequences were <1% distant under a codon-model based synonymous distance estimated using maximum likelihood. This 1% cut-off was chosen based on previous studies [22] and by inspecting the distribution of pairwise distances between putatively unrelated *pol* sequences (subtype B) available from the Los Alamos HIV database (http://www.hiv.lanl.gov/). Importantly, an edge between two nodes does not indicate direct transmission between two individuals, only a close association. Therefore, nodes may be connected through an edge even though the individuals represented may never have had physical contact. Also, the edges in our network are undirected.

### Clinical trial simulations

**Figure 1:**
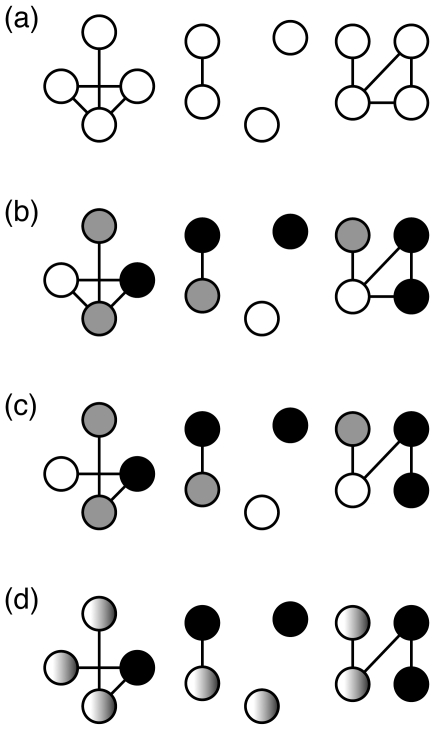
Procedure for simulation of clinical trial on mock network. The clinical trial depicted here has a treatment efficacy of 25% (3 participants on treatment, shown in white, versus 4 on placebo, shown in gray) with an edge removal rate of 40% (2 out of 5 edges connected to infected treatment cases). (a) First, the network is constructed using a synonymous sequence divergence cut-off. (b) Next, trial status is assigned: treatment in white, placebo in gray, and community members in black. Treatment nodes are infected at a reduced rate reflecting treatment efficacy. (c) Edges are then removed from treatment nodes at a given rate to represent the reduction in transmission due to delay in infection and reduction in forward transmission. The metrics are then calculated. The Network Statistic metric is 6 for treatment and 9 for placebo; the Number Infected Statistic is 3 for treatment and 4 for placebo. (d) Finally, a permutation test to determine significance is performed on the modified network by randomizing the assignment of treatment and placebo. Community nodes remain unaltered by the permutation.

We assumed that our simulated clinical trials contained 10,000 participants (5000 in each arm) who become infected at a given rate (incidence rate  = 0.02 infections per length of trial [2], [6], [7]) (Figure 1a). In the absence of an effective intervention, we expect an average of 100 infected individuals in each arm. To simulate a clinical trial of a given duration, we first sampled the number of infected individuals in the treatment (T) and placebo (P) arms using the binomial distribution, where the incidence in the treatment arm was reduced by the assumed treatment efficacy. Next, T random nodes in the network were assigned treatment arm, P – the placebo arm, and the remainder (N minus T minus P) – to infected community members who were not participating in the trial (Figure 1b). A proportion of edges connecting to treatment nodes were removed to approximate (i) the reduction of the number of opportunities for future transmission resulting from the delay in initial infection due to treatment and/or (ii) the reduction in the probability of future transmission of the virus due to efficacious treatment (Figure 1c).

Clinical trials were simulated for: (i) treatments with varying efficacy (i.e. prevention/delay of infection: 0–40%), corresponding to the reduction of the number of nodes in the treatment arm, and (ii) varying efficacy of preventing future infection (i.e. reduced transmissibility/delay of initial infection: 0–100%), corresponding to the reduction in the number of edges connecting to the nodes on treatment. For each scenario, 10,000 clinical trials were simulated, and at the end of each trial, the statistical power of two metrics used to gauge the efficacy of treatment was compared.

The first metric (referred to as the Number Infected Statistic) used the number of infected participants in each arm. The difference between the number of infected individuals in the treatment and placebo arms was calculated. To assess the significance of this difference, we calculated the null distribution of the Number Infected Statistic by performing permutation tests in which the status (treatment versus placebo) of each infected trial participant, represented by a node, was assigned randomly, as if treatment were ineffective, and the difference between the number of infected treatment and placebo nodes was calculated. Treatment status was permuted 10,000 times to construct the null distribution with α = 0.05 (Figure 1d). Note that this procedure is a permutation-based contingency table test, whereas the null model estimated the (unknown) incidence by the observed incidence for the entire trial.

The second metric (referred to as the Network Statistic) makes use of the degree of nodes (the number of edges connected to a given node). Each node (representing an infected trial participant) was given a score: the degree of that node plus 1, to account for the fact that the study participant was infected. Therefore, in the absence of any network connections, the Network Statistic is identical to the Number Infected Statistic. The difference between the cumulative scores of infected individuals in the treatment and placebo arms was calculated. To assess the significance of this difference, we calculated a null distribution by performing a permutation test in the same manner as described above (Figure 1d). The procedure was implemented in Python. The code and the anonymized network structure for the San Diego cohort can be downloaded from http://www.hyphy.org/pubs/NetworkStats/.

## Results

**Figure 2:**
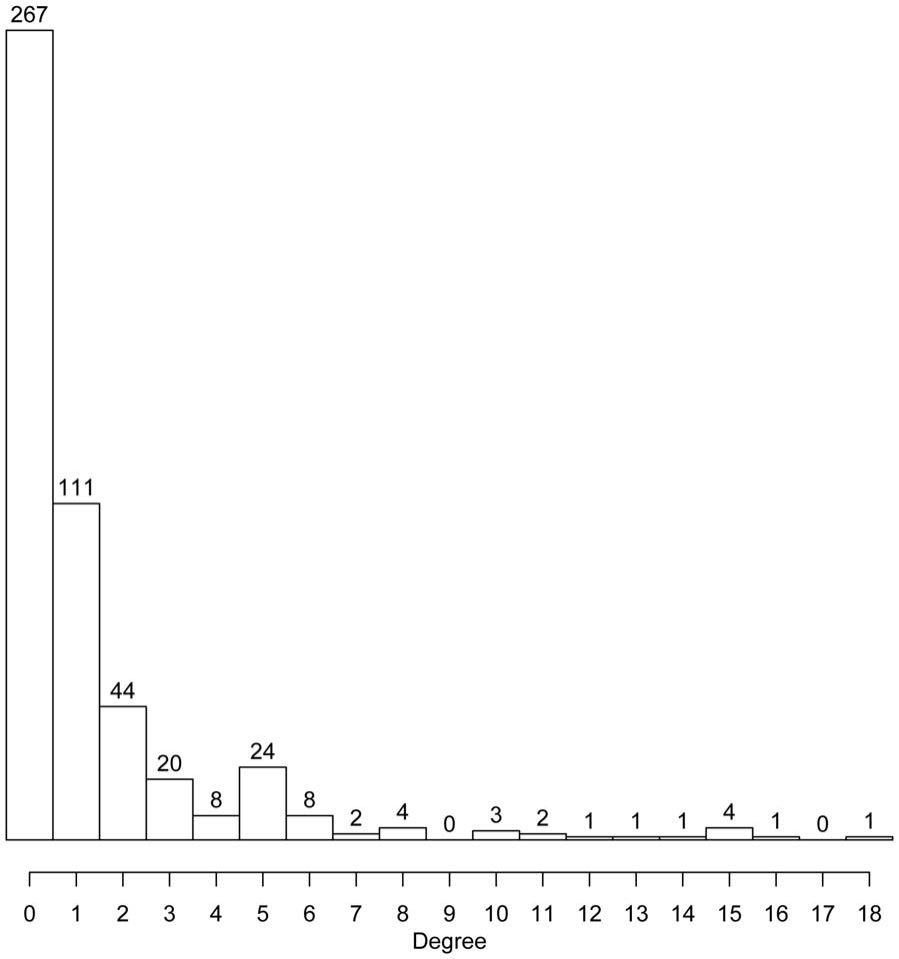
Histogram depicting the distribution of degree across nodes in the San Diego network.

The inferred San Diego network was relatively sparse, containing only 345 edges on 502 nodes when using a 1% synonymous divergence cut-off. Slightly less than half of the nodes (47%) had a degree of one or greater (i.e. at least one edge connected to the node) (Figure 2). The mean degree for all nodes was 1.37, and the maximum degree for a node in the network was 18.

We simulated clinical trials on the HIV transmission network, inferred from the San Diego MSM population, in which the efficacy of treatment varied between 0 and 40%. Using these simulated clinical trials, the statistical power of the Number Infected and the Network Statistics was assessed. We also allowed for the possibility that the intervention both prevented infection and reduced the potential for onward transmission of the virus over a varying range of efficacies, as might arise from the use of PrEP and ART.

**Figure 3:**
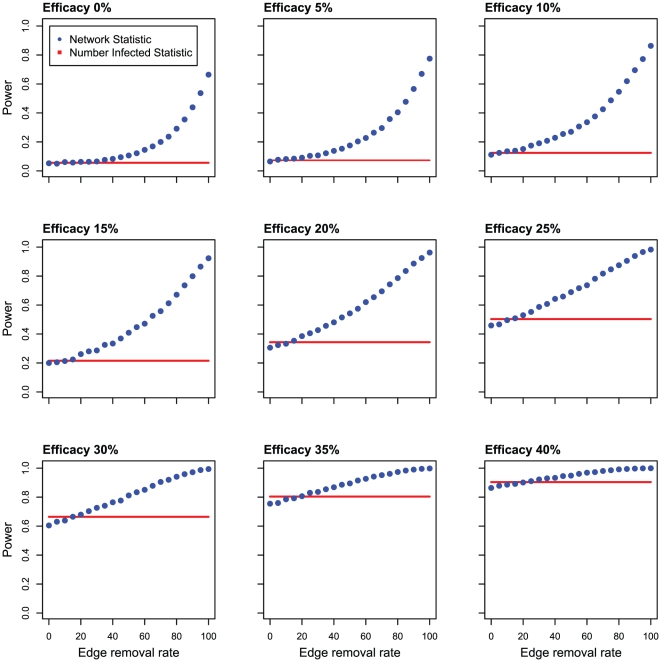
Statistical power of the Network Statistic on simulated clinical trials as function of the edge removal rate on the San Diego network. The Network Statistic values are blue dots. The power of the Number Infected Statistic for a given efficacy is a solid red line.

At every level of intervention efficacy examined, the Network Statistic demonstrated the potential for increased statistical power compared with the Number Infected Statistic (Figure 3). The actual increase in statistical power depended on the proportion of edges removed from each node undergoing treatment due to either a (i) delay in initial infection or (ii) reduction in the probability of future transmission. We note, however, that power gains relative to the Number Infected Statistic were observed only when the number of edges removed exceeded the efficacy of the intervention (i.e. when the number of subsequent infections is reduced to a greater extent than would arise solely from having prevented the infection of the study participant). A plausible example of this scenario would be through a reduction in viral load through ART. The magnitudes of the potential gains in statistical power using the Network Statistic were greatest when the treatment efficacy was low, since the Number Infected Statistic becomes well powered (≥80%) when treatment efficacy reached 35%. Importantly, even though our inferred network was relatively sparse and did not represent the complete transmission network, we still observed the potential for substantial gains in statistical power. Importantly, the Network Statistic and its permutation test preserved the Type-I error rate – the possibility of incorrectly rejecting the null (see Figure 3: 0% efficacy, 0% edge removal rate).

**Figure 4:**
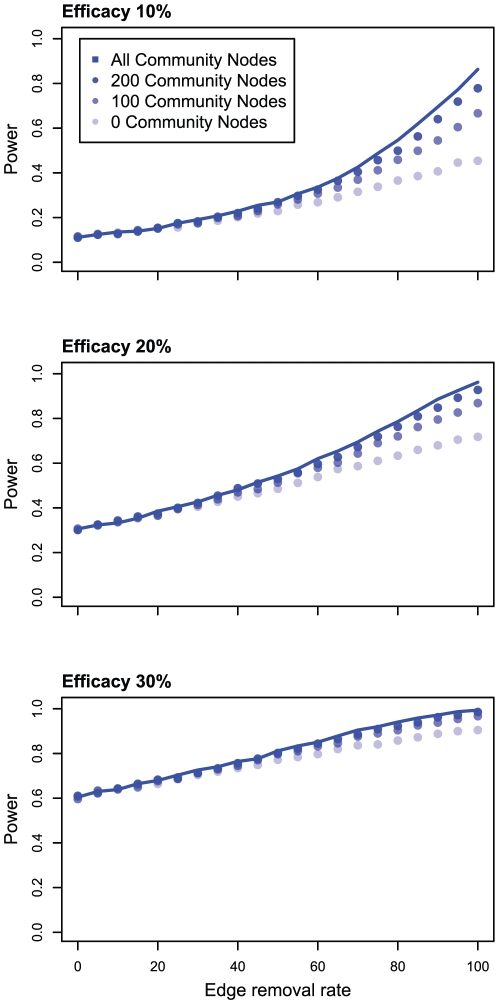
Statistical power of the Network Statistic as a function of the edge removal rate and the number of community nodes. Treatment efficacies of 10%, 20%, and 30% are shown. ‘All community nodes’ corresponds with the power of the Network Statistic in Figure 3.

Next, we explored the impact of the number of community nodes, representing infected individuals who were not study participants, on statistical power. We simulated clinical trials with limited community sampling (e.g. 0, 100, and 200 community nodes). In an actual clinical trial, these sequences would be obtained from individuals who became infected concurrently with the clinical trial in the same geographical region. The reduction in the number of community nodes resulted in a lower statistical power than did trials simulated using the more densely sampled network (Figure 4). Similar to the above simulations, this difference was more pronounced at lower levels of efficacy; however, the decrease in statistical power associated with smaller numbers of community nodes was not evident at low levels of edge removal. This relative unimportance of community nodes at low levels of edge removal may be the result of the sparseness of our network, since most nodes did not share edges with any other nodes, community or otherwise.

**Figure 5:**
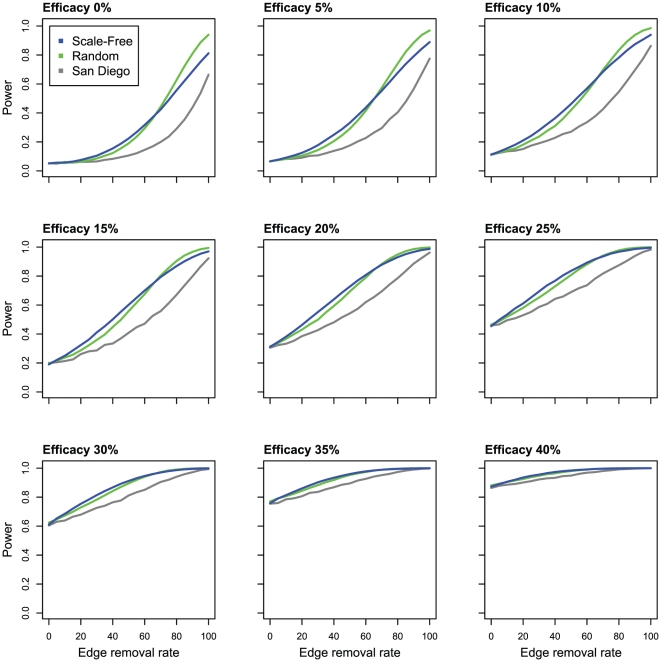
Statistical power of the Network Statistic on simulated clinical trials on simulated networks. Scale-free networks are shown in blue, random networks in green, and the San Diego network in gray. The San Diego network values correspond with the power of the Network Statistic in Figure 3.

Finally, we explored the utility of our Network Statistic on two types of simulated transmission networks: scale-free and random. These simulated networks were constructed to have a similar number of edges as the inferred San Diego network. The statistical power of the Network Statistic was greater on these simulated networks than on the San Diego network (Figure 5). However, when comparing scale-free and random transmission networks, the Network Statistic's power was not consistently better in one versus the other.

## Discussion

These results illustrate potential gains from using network statistics in the analyses of HIV prevention studies. The value is twofold: (i) potential increases in power, particularly if the intervention might have an impact on secondary transmissions from study participants, and (ii) in certain cases, the ability to detect effects that go beyond the study participants themselves and involve the broader community. To assess the actual gains in power using the Network Statistic will require application to PrEP, vaccination, or other prevention studies. Nevertheless, the simulations performed here demonstrate the potential of a simple network-based statistic that accounts for only first-order network interactions; more complicated network statistics (e.g. connectivity) may be even more valuable.

Although accounting for modifications to the network can increase the probability of detecting an effective intervention, one would not expect to achieve the maximum power gains attained here. In practice, unless direction of infection can be ascertained, one would never observe a 100% reduction in edges connected to nodes representing infected study subjects if the network is fully sampled. This is because a source partner node will share at least one edge with the infected recipient (an individual in the treatment arm). Overall statistical power is also likely decreased because many transmission events between study participants and other community members are likely to be missed due to under-sampling and therefore not included in the network. Nonetheless, the permutation tests of the Network Statistic are valid regardless of the amount of genetic information available for recent HIV infections in a community. Additional information would increase the statistical power of the Network Statistic.

An important feature of our Network Statistic, as currently implemented, is its inability to fully distinguish between treatment effects due to infection prevention and those due to transmission prevention. Some insight regarding the importance of both effects can be obtain by comparing the relative magnitudes of the standardized Network Statistic and Number Infected Statistic; standardization could be achieved by obtaining a bootstrap estimate of variance for both statistics. When the Network Statistic, but not the Number Infected Statistic, shows a significant treatment difference, the transmission effect may be of greater importance. In such settings, however, it may still be necessary to combine across effects to achieve a statistically significant result. In any case, a significant result using the Network Statistic would indicate a benefit due to treatment whose exact nature would require further investigation. There are also important settings (e.g. clinical trials investigating test-and-treat interventions, in which all participants are already HIV-positive), where the outcome of interest is exclusively the network-level effect. In such settings, interpretation of the Network Statistic as a measure of the decrease in transmission due to treatment is straightforward.

There are many ways improve the estimation of a transmission network and the associated statistic. First, directionality of edges in the network might be available if estimated dates of infection were observed during the trial or inferred using the results of clinical tests [23] and/or estimates of viral nucleotide diversity [17], [24]. In addition, we could modify the Network Statistic to weight edges by the inverse of the genetic distance between nodes, or some other factor related to the probability of the cluster representing a transmission event. Furthermore, it would be possible to account for the temporal nature of network construction in an actual clinical trial. For the test statistic based on study participants alone, we could use a standard log-rank test. For the Network Statistic, we could calculate, for each person, a value at every observed event time in either arm. This method would provide a counting process for each person that could take on any integer value. From these values, we could calculate a network-based test statistic. For example, for both randomized groups, we could sum the degree over subjects of this process at each event time and then sum the difference between groups over the event times. Calculation of the null distribution of this test statistic could be undertaken using permutation; further work needs to be done to determine the most powerful tests against different specific alternative hypotheses. Finally, agent-based modeling simulations may prove useful in understanding how delay of infection and prevention of transmission relate to the power of the Network Statistic. This type of study would allow for a more practical interpretation of the edge removal rate used in our study. In addition, such simulations will help determine the most appropriate cases for the implementation of our novel Network Statistic.

We note that our methods could apply to any pathogen for which a transmission network can be reconstructed using genetic sequence information (e.g. hepatitis C virus and influenza A virus) and any type of prevention study. Perhaps most interesting would be studies such as PrEP, microbicide, barrier methods, or vaccination where the intervention may impact future transmission by reducing viral load in the source partner or through some other mechanism.
